# Supplementary material for: The Effect of Nutritional Intervention with Lactoferrin, Galactooligosacharides and Vitamin D on the Gut Microbiota Composition of Healthy Elderly Women
Source: Nutrients. 2022 Jun 14;14(12):2468. doi: 10.3390/nu14122468 (PMC9228949; doi:10.3390/nu14122468)
Supplement: Supplementary file 1 [file nutrients-14-02468-s001.zip › nutrients-1751453-supplementary.pdf]

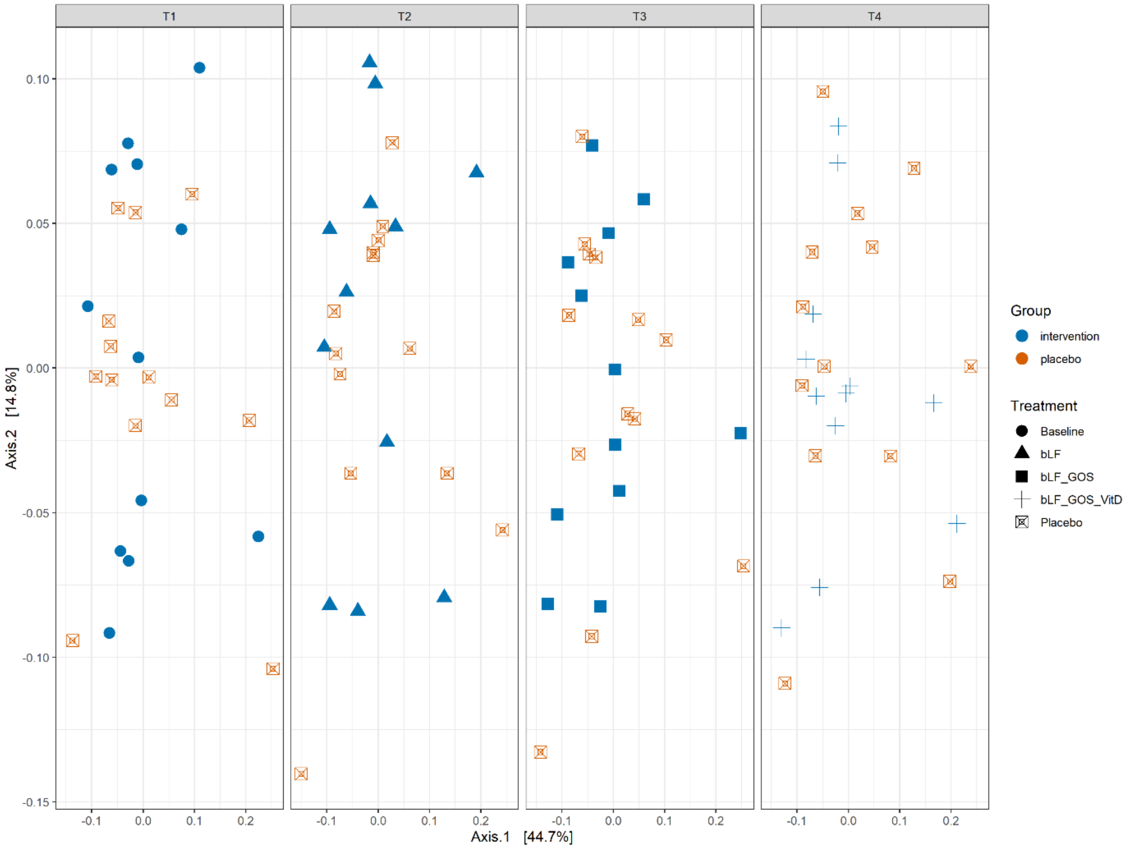

Figure S1. Principal coordinates analysis using weighted UniFrac distances from all the samples included in our study. No differences were observed between the two groups at any of the timepoints tested, as determined by ADONIS analysis of variance with significance set at $p<0.05$. Colours represent the groups each sample belongs to and symbols the corresponding treatment. Percentages at the axes indicate the fraction of observed variation explained.

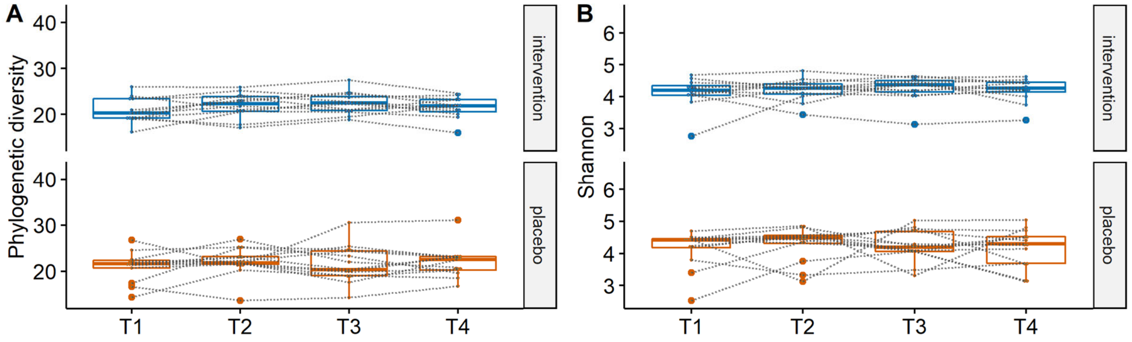

Figure S2. Alpha diversity as determined by the A) phylogenetic diversity, B) Shannon Index and C) Observed OTU's. No significant differences were detected between the two groups. The boundaries of the box on the bottom indicate the 25th percentile, the line within the box represents the median, and the boundary of the box on the top the 75th percentile. No significant differences between or within the groups were detected using Linear mixed effects models.
